# Impact of maternal high‐fat diet on offspring gut microbiota during short‐term high‐fat diet exposure in mice

**DOI:** 10.14814/phy2.70111

**Published:** 2024-11-03

**Authors:** Henry A. Paz, Lasya Buddha, Ying Zhong, James D. Sikes, Umesh D. Wankhade

**Affiliations:** ^1^ Department of Pediatrics, College of Medicine University of Arkansas for Medical Sciences Little Rock Arkansas USA; ^2^ Arkansas Children's Nutrition Center Little Rock Arkansas USA

**Keywords:** diversity, functional profile, gut microbiota, maternal high‐fat diet, offspring, sexual dimorphism

## Abstract

Alterations in the gut microbiome have been linked to obesity, with maternal high‐fat diet (HF) playing a role in shaping offspring microbiome composition. However, the sex‐specific responses to maternal HF diet and the impact of subsequent dietary challenges remain unclear. This study investigated the effects of maternal HF diet on offspring gut microbiota structure and predicted functional profile in response to short‐term postnatal HF diet exposure with a focus on sex‐specific responses. Female and male offspring of maternal control (C) diet or maternal HF diet were weaned onto C diet or HF diet. Offspring were euthanized at 13 weeks of age and cecal contents were collected for bacterial taxonomic profiling. Maternal HF diet reduced α‐diversity, notably in male offspring weaned onto HF diet. Sex‐specific differences were observed in the gut microbial composition and predicted functional potential. Furthermore, the influence of maternal diet on bacterial community structure and functional potential varied depending on postnatal diet. Maternal HF diet led to increased relative abundance of *Corynebacterium* in female offspring and decreased abundance of *Akkermansia* and *Roseburia* in male offspring. These findings underscore the sexually dimorphic nature of maternal HF diet effects on gut microbiota composition and function, with implications for developmental programming and metabolic health.

## Graphical abstract

Maternal high‐fat diet alters offspring gut bacterial community in a sexually dimorphic manner. Maternal high‐fat diet decreased the relative abundance of *Akkermansia* and *Roseburia* in male but not in female offspring. C, control diet; H, high‐fat diet.

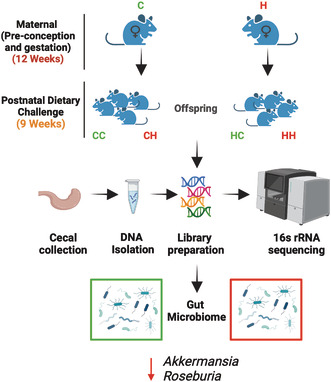

## 1 INTRODUCTION

The prevalence of maternal obesity in the United States is steadily increasing, with prepregnancy obesity ranging between 10.4% and 36.6% (Singh et al., 2021). Moreover, there has been an increase in the percentage of women experiencing excessive weight gain during pregnancy (Leonard et al., 2017). These trends are concerning as obesity during conception and gestation can profoundly impact in utero and early offspring development, programming the offspring's response to postnatal challenges and predisposing them to various metabolic anomalies (Chang et al., 2019; Menting et al., 2019). Both human and animal studies present strong evidence supporting the role of the intrauterine environment on determining disease risk later in life. Our previous research using a rodent model of maternal high‐fat (HF) diet‐induced obesity reinforce these findings, demonstrating that offspring of obese dams exhibit increased obesity, liver steatosis, and altered gut microbiota when exposed to postnatal HF diet challenge (Wankhade et al., 2017, 2018). The expanding body of evidence on developmental programming affecting offspring's weight gain and metabolic traits underscores the vital importance of addressing maternal obesity and its impact on future generations.

The gut microbiome impacts host energy metabolism by supplying fermentation end‐products and modulating metabolic reactions (Heiss & Olofsson, 2018; Martin et al., 2019). Perturbations in the gut microbiome composition have been associated with metabolic disorders including obesity (Boulangé et al., 2016; Muscogiuri et al., 2019). In humans, the abundance of major phyla such as *Actinobacteria* and *Bacteroidetes* decreases, while the abundance of *Firmicutes* and *Fusobacteria* increases in individuals with obesity compared to those at healthy weight (Duan et al., 2021; Ley et al., 2006). Notably, studies have discovered that the maternal diet can significantly influence the development of the offspring's microbiome, which may in turn shape their metabolic responses in both early and later life (Grant et al., 2023; Zou et al., 2023). Maternal HF diet programming leads to alterations in the composition of the gut microbiome. In a nonhuman primate model, maternal HF diet diminished the abundance of the genus *Campylobacter* (Ma et al., 2014). Whereas, in humans, maternal HF diet depleted the abundance of the genus *Bacteroides* (Chu et al., 2016). These results underscore the role of maternal HF diet programming in the reconfiguration of the offspring gut microbiome. However, further clarification is needed to understand the effects of maternal HF diet on the taxonomic structure and diversity of the offspring gut microbiome.

Studies have found sexual dimorphic responses in offspring phenotypes following maternal challenges in utero (Casimiro et al., 2021; Savva et al., 2022; Wankhade et al., 2018). Despite the wide recognition of the importance of offspring sex in developmental programming, the underlying mechanisms are not fully understood. Factors contributing to sexual dimorphism encompass variations in sex hormones, expression of metabolic genes, and body composition (Laaksonen et al., 2003; Wankhade et al., 2017; Wells, 2007). The gut microbiome also shows differences between female and male individuals throughout the lifespan (Valeri & Endres, 2021). Our previous work showed that maternal HF diet influences the bacterial community profile in offspring (Paz et al., 2022). Others have linked maternal obesity with changes in the gut microbiome of toddlers (18–27 months), particularly those from higher socioeconomic backgrounds (Galley et al., 2014). This mounting evidence strongly indicates that the gut microbiome of offspring can be a factor contributing to developmental programming.

Previous studies have reported that maternal HF diet can alter the gut microbial community in offspring at weaning (Xie et al., 2018; Zheng et al., 2022). However, the effects of subsequent postnatal dietary challenges on the gut microbiome remain unclear. In this study, we evaluated the effects of long‐term maternal HF diet on offspring gut microbiota in response to a short‐term postnatal HF diet challenge. We employed a well‐established mouse model of maternal HF diet‐induced obesity, which mirrors low‐quality diets before and during pregnancy and during lactation and is associated with obesity and metabolic dysfunction. We assessed the interactions between maternal diet, offspring diet, and sex of the offspring focusing on their impact on the gut microbiota and predicted functional pathway profiles.

## 2 MATERIALS AND METHODS

### 2.1 Animal experiments

The Institutional Animal Care and Use Committee at the University of Arkansas for Medical Sciences approved all experimental protocols. Four‐week‐old female C57BL6/J mice were purchased from Jackson Laboratories (Bar Harbor, ME) and housed in an AAALAC‐approved animal facility in a temperature‐controlled room (22°C) with a 12 h light:12 h dark cycle. Following 1 week of acclimatization, female mice were fed ad libitum either a control (C) diet (3.8 kcal/g with 18.8% of calories from protein, 63.9% from carbohydrate and 17.2% from fat; TD.95092, Envigo Teklad) or a HF diet (4.7 kcal/g with 14.7% of the calories from protein, 40.7% from carbohydrate and 44.6% from fat; TD.08811, Envigo Teklad) for 12 weeks. At 17 weeks of age, females were bred with 10‐week‐old male mice that were fed the C diet. Offspring remained with birth dams on their respective diets until weaning at 4 weeks of age and then equal number of female (F) and male (M) offspring from both maternal diet groups were randomly assigned to either C or HF diet for 9 weeks. This resulted in eight distinct groups: namely, female offspring of C diet‐fed dams weaned onto C diet (F‐CC) or HF diet (F‐CHF), female offspring of HF diet‐fed dams weaned onto C diet (F‐HFC) or HF diet (F‐HFHF), male offspring of C diet‐fed dams weaned onto C diet (M‐CC) or HF diet (M‐CHF), and male offspring of HF diet‐fed dams weaned onto C diet (M‐HFC) or HF diet (M‐HFHF). Offspring were euthanized at 13 weeks of age by carbon dioxide asphyxiation and cecal contents were collected and immediately snap frozen. Cecal samples were stored at −80°C until analyzed for bacterial taxonomic profiling.

### 2.2 Bacterial community profiling using 16S rRNA amplicon sequencing

Genomic DNA was extracted from the cecal samples using the MO BIO PowerSoil DNA Isolation kit (Qiagen, MD, Catalog # 12955–4) with a few modifications. Cecal contents (20–25 mg) were added directly onto 96‐well plates with beads and recommended buffers in the wells. Sealed plates were shaken horizontally at 20 Hz for 20 min using a mixer mill (Retsch MM 400). The remaining steps were performed according to the manufacturer's protocol. Extracted DNA was quantitated spectrophotometrically and stored at −20°C. Fifty nanograms of genomic DNA were utilized for amplification of the V4 variable region of the 16S rRNA gene using the 515F/806R primers. Forward and reverse primers were dual‐indexed as described by Kozich et al. (2013). Paired‐end sequencing (2 × 250 bp) of pooled amplicons was carried out on an Illumina MiSeq with ~30% PhiX DNA. Raw sequences are available at the NCBI Sequence Read Archive (SRA) under the accession no. PRJNA1128641.

### 2.3 Bioinformatics analysis

Bioinformatics analyses were performed using QIIME2 v2019.7 (Bolyen et al., 2019). Denoising was done with an initial quality filtering (Bokulich et al., 2013) using q2‐quality‐filter followed by the Deblur algorithm (Amir et al., 2017). Amplicon sequence variants (ASV) were aligned with MAFTT (Katoh et al., 2002), and then a phylogenic tree was generated with FastTree (Price et al., 2010). Sampling depth was evaluated using rarefaction curves in conjunction with the Good's coverage index. α‐ and β‐diversity metrics were computed using q2‐diversity. For α‐diversity, observed features, Pielou's index and the Shannon index were used to evaluate richness, evenness and diversity, respectively. For β‐diversity, a principal coordinate analysis (PCoA) using weighted UniFrac distances was conducted (Lozupone et al., 2011). Representative sequences were assigned taxonomy using a Naives Bayes classifier trained on the Silva rRNA database 138 (Quast et al., 2013). Predictions of functional profiles were done using the Phylogenetic Investigation of Communities by Reconstruction of Unobserved States (PICRUSt2) software via q2‐picrust (Douglas et al., 2020).

### 2.4 Statistical analysis

Statistical analyses and figures were performed in R version 4.2.0. α‐diversity indices were assessed using ANOVA and β‐diversity was evaluated using permutational multivariate ANOVA (PERMANOVA) to determine the main effects of maternal diet, offspring diet, and sex, along with their respective interactions. Spearman's rank correlation was used to assess the relationship between genus and pathway abundance. The *p* values were adjusted for multiple testing using the false discovery rate (FDR) method and statistical significance was determined at *p* < 0.05. Bacterial community profiles among samples were depicted in a two‐dimensional PCoA plot using the first two principal component axes. Predicted functional profiles were visualized via a heatmap constructed using ClustVis (Metsalu & Vilo, 2015). The final panel figures were processed using Adobe Illustrator version 26.0.

## 3 RESULTS

### 3.1 Sequencing information

Across samples, quality‐filtered, rarefied reads were clustered into 179 bacterial ASV. For both female and male offspring, rarefaction curves tended to plateau across treatments suggesting sufficient sequencing depth for an adequate characterization of the bacterial community (Figure S1). In addition, the Good's coverage index indicated that at least 97% of the bacterial community was represented across samples. Thus, analyses in this study provided a robust assessment of the cecal bacterial community.

### 3.2 Diversity and taxonomic composition

**FIGURE 1:**
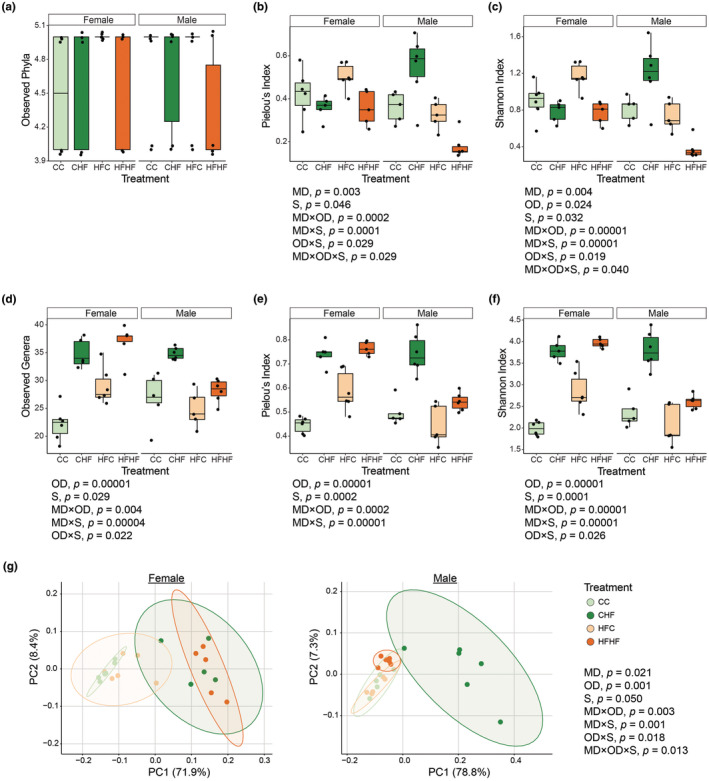
Maternal diet impacts alpha‐ and beta‐diversity metrics in a sex‐dependent manner. Alpha‐diversity metrics including observed features, Pielou's index, and the Shannon index at the (a, b, c) phylum and (d, e, f) genus levels. Two‐dimensional principal coordinate analysis (PCoA) plots based on weighted UniFrac distances for (g) female and (h) male offspring. Numbers in parentheses represent the percent of the variation accounted for the principal‐component axes and ellipses define the 95% confidence level. Three‐way ANOVA and PERMANOVA (maternal diet (MD), offspring diet (OD), sex (S), and interactions) were used to evaluate alpha‐diversity metrics and beta‐diversity, respectively. Pairwise comparisons are presented in Table S1. CC = offspring of control diet‐fed dams weaned onto control diet (female *n* = 6 and male *n* = 5), CHF = offspring of control diet‐fed dams weaned onto high‐fat diet (female *n* = 5 and male *n* = 6), HFC = offspring of high‐fat‐fed dams weaned onto control diet (female *n* = 6 and male *n* = 5), HFHF = offspring of high‐fat‐fed dams weaned onto high‐fat diet (female *n* = 5 and male *n* = 6).

To assess the role of maternal diet on offspring gut microbiota when challenged with a postnatal HF diet, we evaluated α‐ and β‐diversity metrics of the cecal bacterial community. At the phylum level, the observed phyla were similar across treatments for both female and male offspring (Figure 1a). Evenness and the diversity were similar across treatments in female offspring. However, maternal diet impacted evenness and diversity in male offspring weaned onto the HF diet but not the C diet (Figure 1b,c; MD × OD × S, *p* < 0.05). In male offspring weaned onto the C diet, evenness and diversity were similar between offspring of C and HF diet‐fed dams, whereas, in male offspring weaned onto the HF diet, evenness and diversity were lower in offspring of HF diet‐fed dams compared to those of C diet‐fed dams. At the genus level, maternal diet did not impact observed genera in female offspring, whereas male offspring of maternal HF diet had lower number of observed genera compared to those maternal C diet (Figure 1d; MD × S, *p* < 0.001). Evenness and diversity were similar in female offspring of both maternal diets, while male offspring of maternal HF diet exhibited lower evenness and diversity compared to those of maternal C diet (Figure 1e,f; MD × S, *p* < 0.001). To assess β‐diversity, phylogenetic distances (UniFrac) were evaluated using a PCoA. The cecal bacterial community profiles differed between female and male offspring (Figure 1g, p < 0.05). Moreover, the effect of maternal diet on bacterial community composition was sex dependent. For female offspring, bacterial communities differed between maternal diets when offspring were weaned onto the C diet, but not the HF diet. Conversely, in male offspring, bacterial communities varied depending on maternal diets when offspring were weaned onto the HF diet, but not the C diet (MD × OD × S, *p* < 0.05).

**FIGURE 2:**
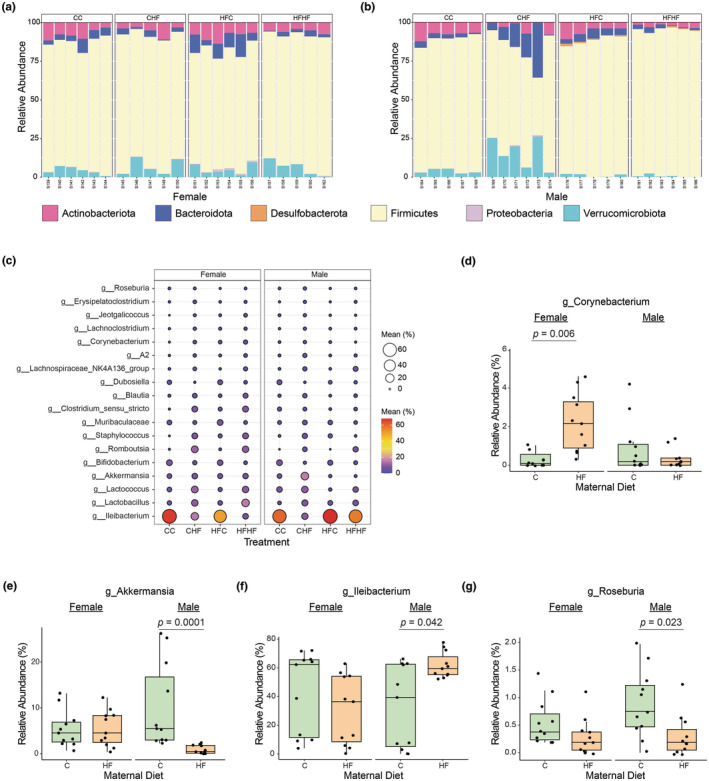
Sexual dimorphic effect of maternal diet on phylum and genus level profiles. Stacked bar chart of the relative abundance at phylum level from the cecal bacterial communities of murine (a) female and (b) male offspring. (c) Bubble plot of the relative abundance at genus level from the cecal bacterial communities of murine female and male offspring. Circle size is proportional to the average relative abundance. Genera with relative abundance >0.5% are represented. (d–g) Maternal diet effect at genus level on female and male offspring. C = control diet, HF = high‐fat diet. *p* values from the Wilcoxon rank sum test.

*Firmicutes* was the most abundant phylum across samples accounting for 82.8% of the total quality‐filtered reads (Figure 2a,b). The remaining phyla included *Actinobacteria* (5.84%), *Verrucomicrobiota* (5.50%), *Bacteriodota* (5.39%), *Proteobacteria* (0.23%), and *Desulfobacterota* (0.14%). Further evaluation revealed a sexual dimorphic effect of maternal diet on the relative abundance of specific phylum (Figure S2). Female offspring of maternal HF diet exhibited a greater relative abundance of *Proteobacteria* phylum in their gut microbiota compared to those from maternal C diet. While male offspring of maternal HF diet showed greater relative abundances of the *Desulfobacteria* and *Firmicutes* phyla, along with lower relative abundances of the *Proteobacteria* and *Verrucomicrobiota* phyla. The predominant genus across samples was *Illeibacterium*, representing 42.1% of the total quality‐filtered reads (Figure 2c). Other main genera were *Lactobacillus*, *Lactococcus*, *Akkermansia*, and *Bifidobacterium* (6.67, 6.49, 5.50 and 4.32% of the total quality‐filtered reads, respectively). Taxonomic composition at the genus level displayed a sexual dimorphic response to maternal diet. The relative abundance of *Corynebacterium* was higher in female offspring of maternal HF compared to maternal C diet, while there was no difference in male offspring (Figure 2d). The relative abundance of *Akkermansia* and *Roseburia* was lower and of *Ileibacterium* was higher in male offspring of maternal HF diet compared to maternal C diet (Figure 2e–g). No differences were observed between maternal diets in female offspring regarding these genera. Interestingly, the lower relative abundance of *Ileibacterium* in male offspring of maternal C diet was primarily driven by feeding HF diet postnatally (Figure 2c).

**FIGURE 3:**
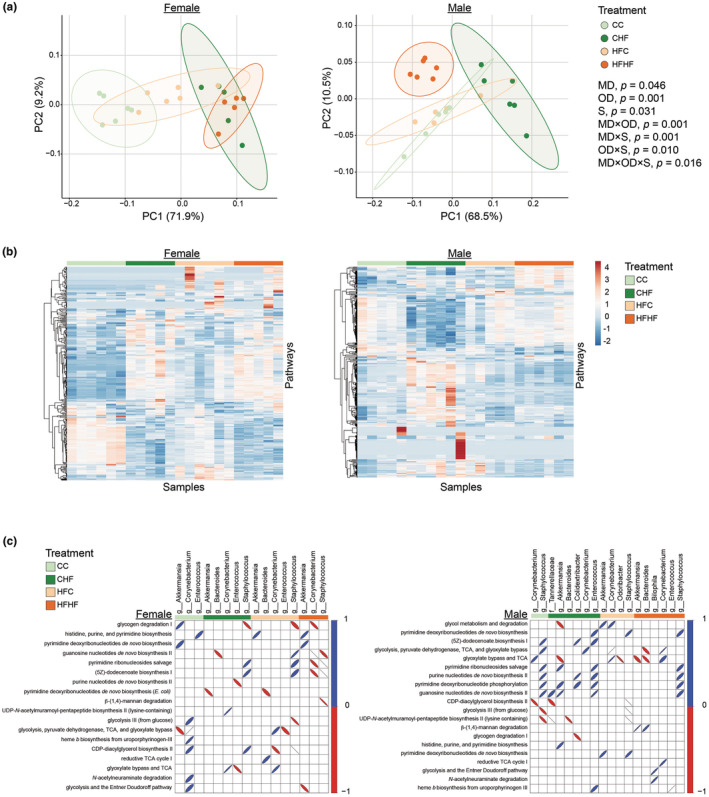
Effects of maternal diet on predicted functional profiles differ by sex. (a) Two‐dimensional principal coordinate analysis (PCoA) plot based on Bray‐Curtis dissimilarities from PICRUSt2‐predicted functional profiles. Numbers in parentheses represent the percent of the variation accounted for the principal‐component axes and ellipses define the 95% confidence level. (b) Heatmaps displaying distinct functional profiles. (c) Correlations between genus and pathway abundances. Three‐way PERMANOVA (maternal diet (MD), offspring diet (OD), sex (S), and interactions). Pairwise comparisons are presented in Table S1. CC = offspring of control diet‐fed dams weaned onto control diet (female *n* = 6 and male *n* = 5), CHF = offspring of control diet‐fed dams weaned onto high‐fat diet (female *n* = 5 and male *n* = 6), HFC = offspring of high‐fat‐fed dams weaned onto control diet (female *n* = 6 and male *n* = 5), HFHF = offspring of high‐fat‐fed dams weaned onto high‐fat diet (female *n* = 5 and male *n* = 6).

PICRUSt2 was used to predict the functional capabilities of the gut microbial community. Differences in bacterial community structure between female and male offspring led to distinct predicted functional profiles (Figure 3a; S, *p* < 0.05). Furthermore, the influence of maternal diet on functional profiles showed a sex‐specific pattern. In female offspring, disparities in functional profile were evident between maternal diets upon weaning onto the C diet, whereas no difference was observed upon weaning onto the HF diet. On the contrary, in male offspring, functional profiles exhibited variations based on maternal diet upon weaning onto the HF diet, but not the C diet (MD × OD × S, *p* < 0.05). Additional evaluation of functional profiles using heatmaps supported sex‐specific responses to maternal HF diet (Figure 3b). In both female and male offspring, significant correlations were identified between the genera *Akkermansia*, *Bacteroides*, *Corynebacterium*, *Enterococcus*, and *Staphylococcus* and distinct predicted metabolic functions (Figure 3c). Noteworthy, in genera impacted by maternal diet different correlation patterns were observed. In female offspring of maternal C diet, *Akkermansia* and *Corynebacterium* were positively correlated with pathways involved in the generation of energy, whereas in female offspring of maternal H diet, these genera were positively correlated with pathways involved in nucleoside and nucleotide biosynthesis. Conversely, in male offspring of maternal H diet, positive correlations were observed between *Akkermansia* and *Corynebacterium* and energy and degradation pathways.

## 4 DISCUSSION

Dysbiosis in the gut microbial community has been linked to obesity and related co‐morbidities (Aron‐Wisnewsky et al., 2021). This study aimed to evaluate the effect of maternal HF diet on offspring gut microbiota in response to short‐term postnatal HF diet exposure. Maternal HF diet altered α‐ and β‐diversity in a sex‐specific manner. Intriguingly, the influence of maternal diet on bacterial community structure and functional potential varied depending on postnatal diet. For the composition of the gut microbiota, maternal HF diet induced dysregulation of the community in a sex‐dependent manner by increasing the relative abundance of the genus *Corynebacterium* in female offspring and reducing the relative abundance of the genera *Akkermansia* and *Roseburia* in male offspring. These alterations to the gut microbiome could potentially contribute to sex‐specific developmental programming effects.

Maternal HF diet has been shown to reduce α‐diversity in offspring during early life stages, but this decline tends to gradually recover as the offspring age (Xie et al., 2018). In this study, maternal HF diet affected α‐diversity in a sex‐specific manner, resulting in a decrease in male offspring but not in female offspring. Interestingly, a negative additive effect on α‐diversity was observed in male offspring of maternal and postnatal HF diets at higher taxonomic level (phylum), but this effect did not extend to lower taxonomic level (genus). Previous research from our group showed no effect of maternal HF diet on α‐diversity in male offspring (Paz et al., 2022). However, mice in that study were older (24‐weeks), supporting a potential gradual recovery of α‐diversity as the age of offspring increases. The implications of reduced diversity at early life stages on the trajectory of the bacterial community composition and its effect on the host require further evaluation.

Maternal HF diet has been shown to impact the composition of the gut microbiome in offspring. For instance, a decrease in the abundance of the genera *Akkermansia* and *Lactobacillus* has been reported (Schneeberger et al., 2015; Zheng et al., 2022). At the phylum level, we observed an increase in the abundance of *Desulfobacteria* and *Firmicutes* in offspring of maternal HF diet. Both phyla include pathogenic members and have been associated with obesity (Yang et al., 2023; Zhang et al., 2023). At the genus level, we observed a marked reduction in the abundance of *Akkermansia* due to maternal HF diet; however, this effect was observed only in male offspring. The genus *Akkermansia* is a common constituent of the human gut microbiota (Lv et al., 2022). The member *A*. *muciniphila* is known for its role as a mucin degrader which contributes to the maintenance of the intestinal barrier integrity (Collado et al., 2007). Furthermore, *A*. *muciniphila* is negatively associated with obesity and related metabolic disorders, prompting its investigation as a promising candidate for mitigating these conditions (Cani & de Vos, 2017; Schneeberger et al., 2015). In the current study, maternal HF diet also decreased the abundance of the genus *Roseburia* in male but not female offspring. Members of the genus *Roseburia* are predominant butyrate producers involved in functions related to energy metabolism, gut barrier integrity and motility, and anti‐inflammation (Nie et al., 2021; Tamanai‐Shacoori et al., 2017). Moreover, *Roseburia* is considered a potential marker of gut health (Tamanai‐Shacoori et al., 2017). We also noted a sex‐dependent response to maternal HF diet in the abundance of *Ileibacterium*. Specifically, there was an increase in the abundance of *Ileibacterium* in male offspring of maternal HF compared to maternal C, whereas no significant change was observed in female offspring. *Ileibacterium* is a recently described genus belonging to the family Erysipelotrichaceae (Cox et al., 2017). Compared to baseline, den Hartigh et al. reported a gradual decrease in the abundance of *Ileibacterium* at 4 and 8 weeks when a high‐fat, high‐sucrose diet was restricted to 75–85% ad libitum and mice lost 14.5% of baseline body weight (den Hartigh et al., 2018). There is limited information regarding the effects of maternal HF diet exposure on the abundance or functions of *Ileibacterium* in offspring. However, our results demonstrate a notable and consistent increase in abundance from this genus in male mice of maternal HF diet. Taken together, these findings suggest that *Ileibacterium* species may play a role in obesity. In female mice, offspring of maternal HF diet had an increase in the abundance of the genus *Corynebacterium*. While some members of this genus are described as skin commensals, others can act as opportunistic pathogens and produce virulence factors (Bernard, 2012; Trost et al., 2011). Overall, our findings show that maternal diet influences the gut microbiota trajectory, leading to a sexually dimorphic dysbiosis.

Sexual dimorphism of the gut microbiome has been reported in animal models (McGee & Huttenhower, 2021). Several factors that have been linked to this response, including sex hormones, genetic background, diet, environmental factors, and bile acids. For instance, the gut microbiome composition changes after puberty and is influenced by androgens (Yurkovetskiy et al., 2013; Gao et al., 2021). Research in mice has demonstrated sex‐dependent differences in the gut microbiota composition across strains, indicating a genetic influence on the microbiota profile (Org et al., 2016). Additionally, bile acids can affect the gut microbiota through their antimicrobial properties, with bacterial families such as S24‐7, Lachnospiraceae, and Streptococcus being associated with deoxycholic acid and taurodeoxycholic acid (Wankhade et al., 2018). In the current study, the bacterial community structure differed by sex. Interestingly, the influence of maternal HF diet on β‐diversity was modulated by postnatal diet in both female and male mice. These results highlight the importance of dietary influence on the structural composition of the gut microbial community. Others have also reported alterations on the overall microbial community structure by maternal HF diet (Di Gesù et al., 2022; Xie et al., 2018); however, our results further demonstrate effects on the bacterial community composition by postnatal dietary challenges. The predicted functional profile also displayed sexually dimorphic responses to maternal HF diet. In male offspring, abundant genera affected by maternal HF diet showed positive correlations with energy and degradation pathways, whereas in femal offspring, these were positively correlated with nucleoside and nucleotide biosynthesis pathways. Disparities in the metabolic potential of the gut bacterial communities may contribute to the programming effects of maternal HF diet.

Several limitations from this study should be noted. First, extrapolating findings from mouse studies to humans require caution due to inherent physiological, metabolic, and genetic differences that can affect the applicability of the results (Masopust et al., 2017; Rivera & Tessarollo, 2008). Second, the accuracy of the amplicon‐based function prediction tool PICRUSt2 depends on reference genomes and its resolution is based on the marker gene's ability to resolve different taxa. Third, biological mechanisms driving sex‐specific responses require further exploration. Despite these limitations, our findings highlight the sexually dimorphic effect of maternal HF diet on the gut microbiota composition and function. In summary, our data showed that maternal HF diet impacts the gut bacterial community of offspring in a sexually dimorphic manner. This dysbiosis was characterized by a decreased α‐diversity and shifts in β‐diversity, resulting in alterations in the gut bacterial community structure and metabolic potential. In female offspring, there was an increase in the relative abundance of the genus *Corynebacterium*, whereas in male offspring, there was a decrease in the relative abundance of the genera *Akkermansia* and *Roseburia*. Furthermore, maternal HF diet influenced the bacterial community responses to postnatal dietary challenges. Overall, these modifications to the gut microbiome may contribute to developmental programming in a sex dependent manner.

## AUTHOR CONTRIBUTIONS

UDW conceptualized the study; UDW, HAP, LB, JDS, and YZ conducted the experiments; HAP, UDW, and LB performed the data analysis; and HAP, UW, and LB wrote and edited the manuscript. All authors have read and agreed to the published version of the manuscript.

## FUNDING INFORMATION

This research was funded in part by the United States Department of Agriculture‐Agricultural Research Service Project 6026–51,000‐010‐05S. UW is also supported by the Arkansas Children's Research Institute, the Arkansas Biosciences Institute, and the Center for Childhood Obesity Prevention funded by the National Institutes of Health (P20GM109096). Manuscript preparation was also aided by the guidance provided by “Writing Block,” funded by the Center for Childhood Obesity Prevention through the National Institute of General Medical Sciences of the National Institutes of Health under Award Number P20GM109096. UW also received technical assistance from the National Center for Advancing Translational Sciences of the National Institutes of Health under award number UL1 TR003107.

## CONFLICT OF INTEREST STATEMENT

The authors declare that they have no perceived or potential conflicts of interest, financial or otherwise.

## ETHICS STATEMENT

The studies involving mice were reviewed and approved by The Institutional Animal Care and Use Committee at the University of Arkansas for Medical Sciences.

## Supporting information


Figure S1.

Table S1.

## Data Availability

Raw sequences are available at the NCBI Sequence Read Archive (SRA) under the accession no. PRJNA1128641.
