# Supplementary material for: Impact of maternal high‐fat diet on offspring gut microbiota during short‐term high‐fat diet exposure in mice
Source: Physiol Rep. 2024 Nov 3;12(21):e70111. doi: 10.14814/phy2.70111 (PMC11531878; doi:10.14814/phy2.70111)
Supplement: Supplementary file 1 — Figure S1. [file PHY2-12-e70111-s002.pdf]

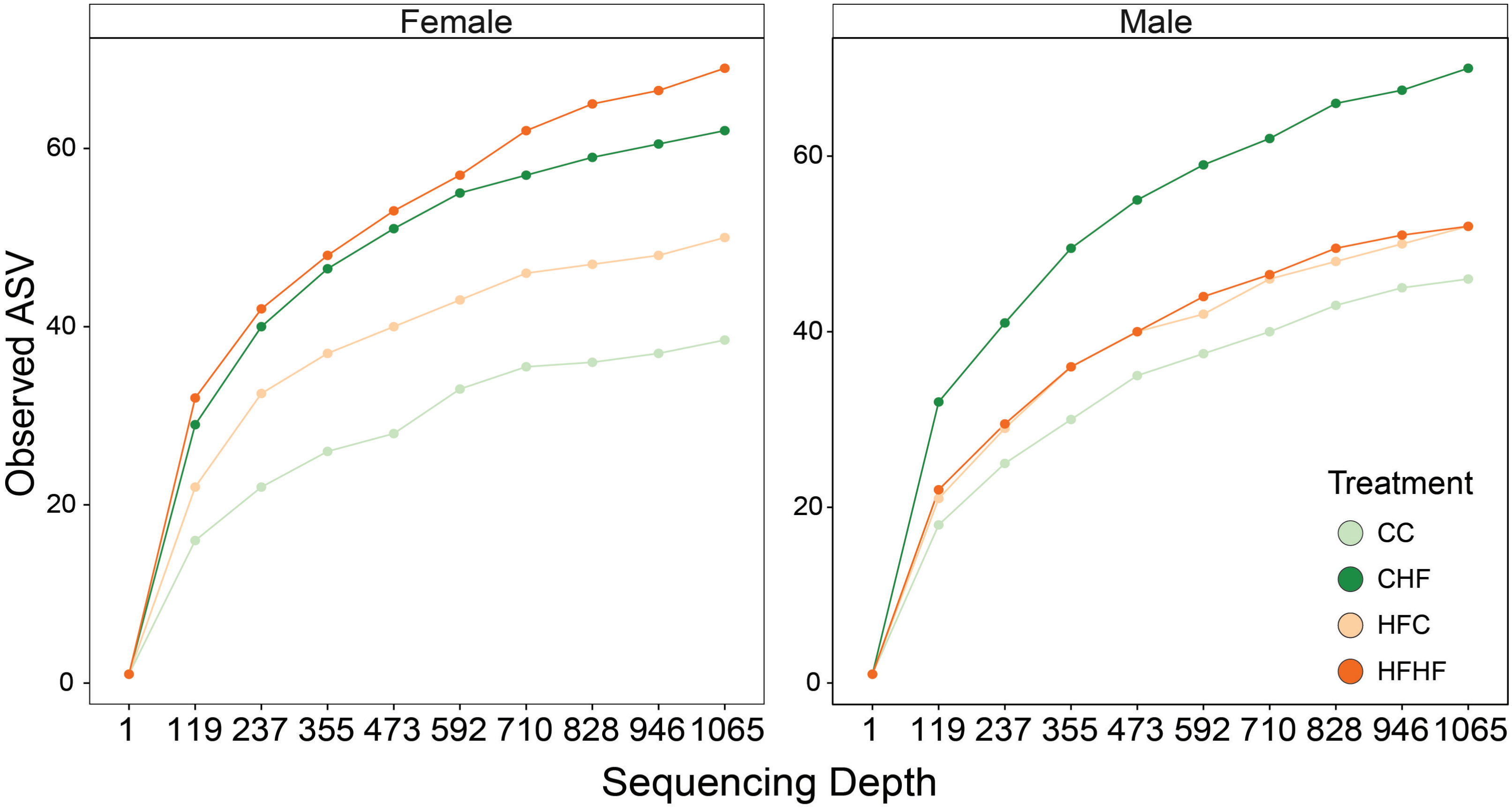


**Supplementary Figure S1**

Rarefaction curves across treatments. Each point represents the median value from 10 iterations. CC = offspring of control diet-fed dams weaned onto control diet, CHF = offspring of control diet-fed dams weaned onto high-fat diet, HFC = offspring of high-fat-fed dams weaned onto control diet, HFHF = offspring of high-fat-fed dams weaned onto high-fat diet.

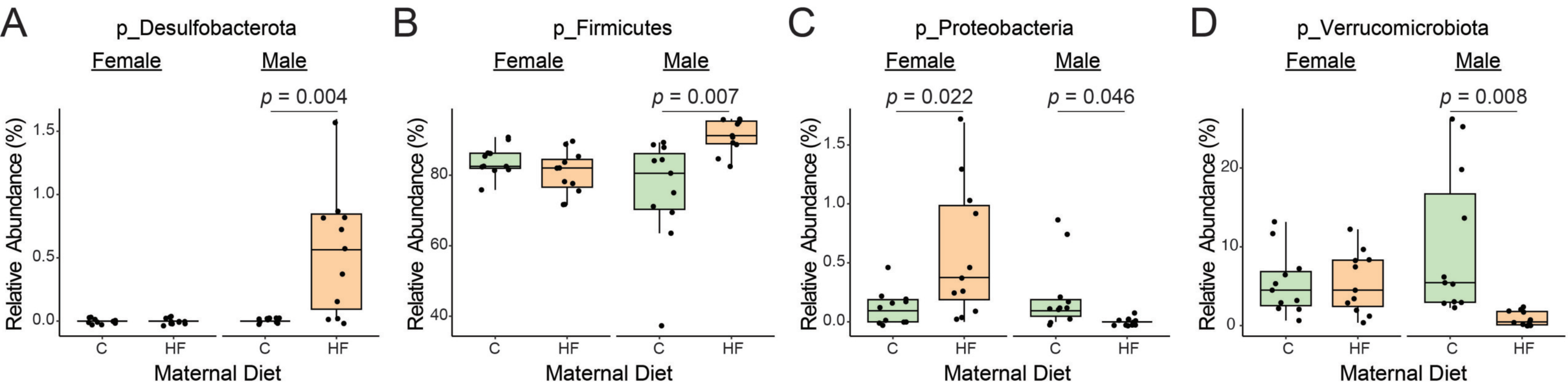


**Supplementary Figure S2**

Sexual dimorphism in gut microbiota composition at the phylum level in response to maternal high-fat diet. C= control diet, HF=high-fat diet.
